# Fabrication of an Electrochemical Sensor Based on Gold Nanoparticles/Carbon Nanotubes as Nanocomposite Materials: Determination of Myricetin in Some Drinks

**DOI:** 10.1371/journal.pone.0096686

**Published:** 2014-05-08

**Authors:** Reza Hajian, Nor Azah Yusof, Tayebe Faragi, Nafiseh Shams

**Affiliations:** 1 Institute of Advanced Technology, University Putra Malaysia, Serdang, Selangor, Malaysia; 2 Department of Chemistry, Faculty of Science, University Putra Malaysia, Serdang, Selangor, Malaysia; 3 Department of Chemistry, Faculty of Science, Gachsaran Branch, Islamic Azad University, Gachsaran, Iran; Macau University of Science and Technology, Macau

## Abstract

In this paper, the electrochemical behavior of myricetin on a gold nanoparticle/ethylenediamine/multi-walled carbon-nanotube modified glassy carbon electrode (AuNPs/en/MWCNTs/GCE) has been investigated. Myricetin effectively accumulated on the AuNPs/en/MWCNTs/GCE and caused a pair of irreversible redox peaks at around 0.408 V and 0.191 V (vs. Ag/AgCl) in 0.1 mol L^−1^ phosphate buffer solution (pH 3.5) for oxidation and reduction reactions respectively. The heights of the redox peaks were significantly higher on AuNPs/en/MWNTs/GCE compare with MWCNTs/GC and there was no peak on bare GC. The electron-transfer reaction for myricetin on the surface of electrochemical sensor was controlled by adsorption. Some parameters including pH, accumulation potential, accumulation time and scan rate have been optimized. Under the optimum conditions, anodic peak current was proportional to myricetin concentration in the dynamic range of 5.0×10^−8^ to 4.0×10^−5^ mol L^−1^ with the detection limit of 1.2×10^−8^ mol L^−1^. The proposed method was successfully used for the determination of myricetin content in tea and fruit juices.

## Introduction

At present, there has been an increasing usage of nano-materials and their applications in analytical chemistry due to their physicochemical characteristics. Gold nanoparticles possess some good properties, as quantized charging/discharging, conductivity, catalytic and photocatalytic activity [1]–[4]. Voltammetric sensors based on gold nanoparticles for determination of biological molecules have received much interest due to their good stability and biocompatibility [5]. Carbon nanotubes (CNTs) are another novel nano-material, which have captured worldwide researchers' interests since their discovery in 1991 [6]. CNTs have ability to hold the potential for wide applications in electrochemistry due to their small dimensions, high surface area, high electrical conductivity, unique structures, significant mechanical strength and good chemical stability [7]. Metal nanoparticles can be immobilized on the solid surface through covalently attached tether layers, and their particle sizes and densities can be independently dominated by pre-synthesizing the nanoparticles and controlling the tether density or assembly conditions [8]. Cruickshank carried out the electrostatic assembly of citrate-capped AuNPs on ethylenediamine (en) tether layers and were electrografted to glassy carbon. It was found that the modified electrode showed a constant activity for reduction of H_2_O_2_ [9].

Flavonoids are polyphenolic benzo-c-pyrone compounds that belong to a class of water soluble plant pigments. More than 6000 different flavonoid molecules have been identified. They occur in vegetables, fruits and beverages like beer, wine, tea and fruit drinks [10]. Flavonoids possess antioxidative, anticancer and cytoprotective properties [11], [12] and have been applied in traditional Chinese medicines successfully for the treatment of depression and anxiety [13]. Myricetin (3,3′,4′,5,5′,7-hexahydroxy flavone), a naturally occurring flavonoid, is classified as a flavonoid with strong antioxidant effects. Oxidative stress plays a key role in various neurological diseases such as ischemia and Alzheimer's disease.

A short ionic liquid based monolithic cartridge was prepared and used as the selective extraction sorbent for myricetin and quercetin [14]. Chromatographic analysis have been conducted on a C_18_ column with UV detection at 372 nm, with an eluting solution consisting of acetonitrile-water (25/75, V/V) as mobile phase, and a flow rate of 0.7 mL·min^−1^. A new approach for the extraction and determination of myricetin and quercetin by using SPME-HPLC-UV system has been developed [15]. The method involves adsorption of flavonoids on a fiber followed by desorption in the desorption chamber of SPME-HPLC interface using citrate buffer (0.001 mol L^−1^): acetonitrile (70∶30) as mobile phase and UV detection at 372 nm.

Nevertheless, some of these methods such as chromatographic methods are time-consuming, expensive, and need complicated preconcentration, multisolvent extraction as well as trained technicians. Compared with other methods, electrochemical methods are characterized by simplicity, high sensitivity, good stability, low-cost instrumentation, small dimensions and on-site monitoring [16]. Up to now, there is no report for determination of myricetin by electrochemical methods. The preparation and application of AuNPs/en/MWCNTs composite film modified glassy carbon electrode for the determination of myricetin in tea and some fruit juices is demonstrated in this study. Experiments revealed that the redox peaks for myricetin can be remarkably enhanced on AuNPs/en/MWNTs/GCE, meaning good electrocatalytic activity and sensitivity for the oxidation of myricetin.

## Experimental

### Reagents and solutions

Myricetin was purchased from Sigma-Aldrich Co. and used as received. Stock solution of myricetin (1.0×10^−3^ mol L^−1^) was prepared in ethanol as solvent and diluted with 0.1 M phosphate medium (pH 3.5) before use. Multi-wall carbon nanotubes (diameter: 10–20 nm, length: 1–2 µm, purity >95%) were obtained from Sigma-Aldrich Co. Gold nanoparticles with average diameters of ∼13 and ∼40 nm were prepared by the reduction of HAuCl_4_.3H_2_O (Merck) with sodium citrate. Other chemicals were analytical grade and used without further purification in double-distilled water.

### Apparatus

Autolab 302N electrochemical system (Metrohm Co., Ltd. Switzerland) was employed for all the voltammetric measurements. A conventional three-electrode system was used, including a bare glassy carbon electrode (GCE) or AuNPs/en/MWNTs film modified GCE as working electrode, a Ag/AgCl (3.0 mol L^−1^ KCl) electrode as reference electrode and a graphite bare electrode as auxiliary electrode. All pH measurements were recorded with a pH meter (Metrohm, model 827).

### Preparation of GC/MWCNTs modified electrode

A bare GCE was pretreated carefully with 0.05 µm alumina slurry on a polishing cloth, rinsed thoroughly with 1∶1 HNO_3_ – H_2_O (v/v), and then washed with pure ethanol and redistilled water, respectively. 10 mg of the untreated MWCNTs was added to a large amount of concentrated nitric acid (wt. 68%), and then sonicated for about 4 h. Then mixture was filtered and washed with doubly distilled water until the filtrate was neutral. The treated MWCNTs were dried under an infrared lamp. MWCNTs suspension was accomplished as follows: 5.0 mg of treated MWCNTs was sonicated in 10.0 ml N, N-dimethylformamide (DMF) for about 30 min after that a homogeneous suspension was obtained. The pretreated GCE was coated evenly with 10.0 µL of MWCNTs suspension, and then DMF was evaporated under an ultraviolet lamp. Before using, the modified electrode was washed repeatedly with double-distilled water to remove loosely bound modifiers [17].

### Citrate-capped Au nanoparticles

Gold nanoparticles (AuNPs) with average diameters of ∼13 and ∼40 nm were prepared by the reduction of HAuCl_4_ with sodium citrate following the literature method [18]. All glasswares used for the preparation of AuNPs were thoroughly washed with freshly prepared aqua regia (HNO_3_∶HCl) (1∶3), rinsed extensively with ultra high purity water sequentially, and then dried in an oven at 100°C for 2 to 3 h. Then, A 60 mL solution of 0.01% (w/v) HAuCl_4_ was brought to a boil with vigorous stirring in a round-bottom flask fitted with a reflux condenser. Then, different amount of 1.0% (w/v) sodium citrate was added to HAuCl_4_ solution (for 13 nm and 40 nm gold NPs, 4.5 and 0.6 mL of sodium citrate were used, respectively). The reaction was maintained at the boiling point with continuous stirring for about 15 min. Then a red color suspension was prepared and stored at 4°C until further usage.

### Modification of GC/MWCNTs with AuNPs

**Figure 1:**
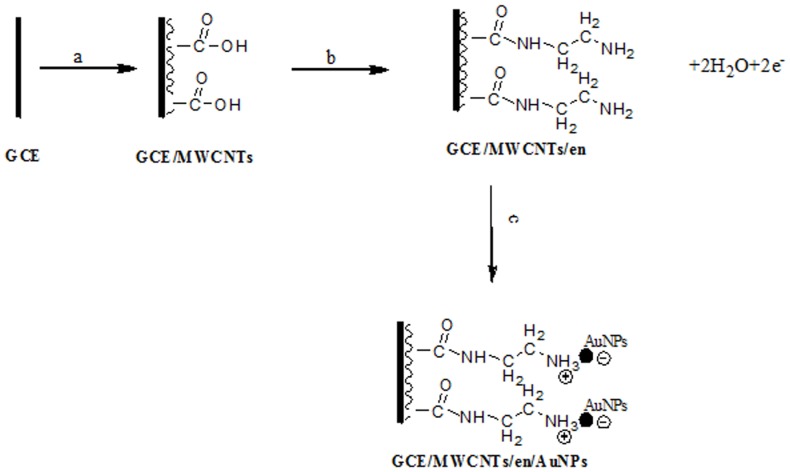
Schematic illustration of the preparation procedure: coating MWCNTs suspension on GCE (a), electrografting en films to MWCNTs/GCE (b), and electrostatic assembly of citrate-capped AuNPs on ethylenediamine (c).

The grafting of primary amines to carbon surfaces by electrooxidation in anhydrous conditions is well-established [19]–[21]. Fig. 1 shows the proposed overall reaction which results in formation of a surface C–N covalent bond. Careful studies have demonstrated that the initially- formed radical cation deprotonates giving a C-centred radical, followed by isomerisation to an amino radical which covalently couples to the surface [21]. Cyclic voltammetric scans of en are consistent with grafting of a blocking film to the GC electrode. There is an irreversible redox process at Epa ∼0.9 V assigned to amine oxidation and on repeat scans the peak current decreases and is absent by the 6th cycle.

Before modification of GC/MWCNTs with AuNPs, ethylenediamine (en) film electrografted on the surface of MWCNTs/GCE as follows: in brief, the potential was cycled six times between 0.0 V to 1.4 V (vs. Ag/AgCl) at 10 mV s^−1^ in a solution of 0.1 mol L^−1^ en-ethanol containing 0.01 mol L^−1^ KClO_4_ as the electrolyte. After electrografting, the surface was rinsed with ethanol, and then followed by double-distilled water and dried with nitrogen gas. To assemble gold nanoparticles, en/MWCNTs/GCE was immersed in citrate-capped AuNPs for 2 h at 4°C in dark. The bonding of AuNPs on amine groups is based on electrostatic assembly as it has shown in Fig. 1. After treatment, the modified electrode was rinsed with double-distilled water, dried with a gentle stream of N_2_ and used immediately.

### Preparation of real samples

Preparation of tea: The preparation consisted of addition of water to tea leaves (1 g) in a 100 mL conical flask and stirred by a magnetic bar on a hot plate at 70°C for 10 min [22]. Then, tea solution was filtered through a Whatman paper (No. 1) and the residue was washed with distilled water (3×10 mL). The prepared tea solution was cooled in room temperature and pH of the solution was adjusted to about 3.5 with the addition of phosphate buffer solution. The final solution was diluted to 250 mL with double distilled water.

Preparation of fruit juices: The juice samples were first filtered and then 1.0 ml was diluted to about 10 ml with distilled water. pH of each filtered solution was adjusted to 3.5 by addition of 1.0 ml phosphate buffer solution prior to analysis [23].

### Analytical Procedure

Unless as otherwise stated, 0.1 mol L^−1^ phosphate buffer solution (pH 3.5) was used as the supporting electrolyte for myricetin determination. An aliquot of the solution containing myricetin was diluted to an appropriate concentration before commencing the voltammetric scan. Before each measurement, the three-electrode system was installed in a blank solution, and cyclic voltammetry scan from −0.2 to 0.8 V (vs. Ag/AgCl) with accumulation potential (E_acc_) of 0.0 V was recorded after an accumulation time (t_acc_) of 60 s on the surface of modified electrode. The quantitative determination of myricetin was achieved by measuring the oxidation peak current after background subtraction using cyclic voltammetry technique.

## Results and Discussion

### Characteristics of AuNPs/en/MWCNTs/GCE

MWCNTs were coated on GCE evenly and used to form pendant chains of ethylenediamine through electrochemical grafting. When ethylenediamine was electrografted using six cycles between 0.0 and 1.4 V (vs. Ag/AgCl) at 10 V s^−1^, there was an anodic peak at approximately 1.05 V in the first cycle, and then the anodic peak almost disappeared in second cycle. This phenomenon is very similar to that in the reported literature [24]. The final step was electrostatic assembly of citrate-capped AuNPs on en tether layers electrografted to MWCNTs (Fig. 1).

**Figure 2:**
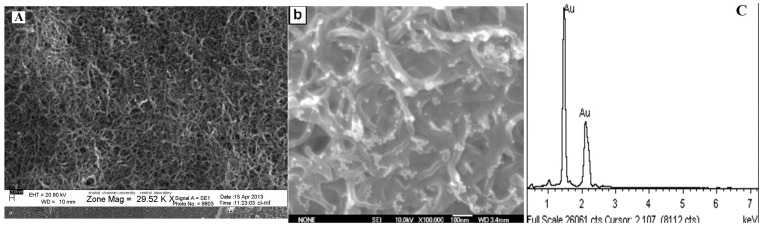
SEM images of MWCNTs film (a) and AuNPs/en/MWCNTs films (b). (c) EDX spectrum of MWCNTs decorated with AuNPs.

Scanning Electron Microscopy (SEM) can effectively prove surface morphologies of the modified electrode. The morphology of MWCNTs film showed a network-like structure (Fig. 2a). Compared with the MWCNTs film, the SEM image of AuNPs/en/MWCNTs films (Fig. 2b) displayed many observable light dots which are due to the assembly of AuNPs. SEM image (Fig. 2b) confirmed that gold nanoparticles are typically bound on MWCNTs with fairly even distribution, although a few aggregates were observed. Fig. 2 C also shows the Energy Dispersive X-ray (EDX) spectrum for the MWCNTs decorated with AuNPs. One can see that the Au signals are quite strong because of the aggregation of AuNPs onto MWCNTs.

### Initial Investigation on the Electrochemical Response of Myricetin

**Figure 3:**
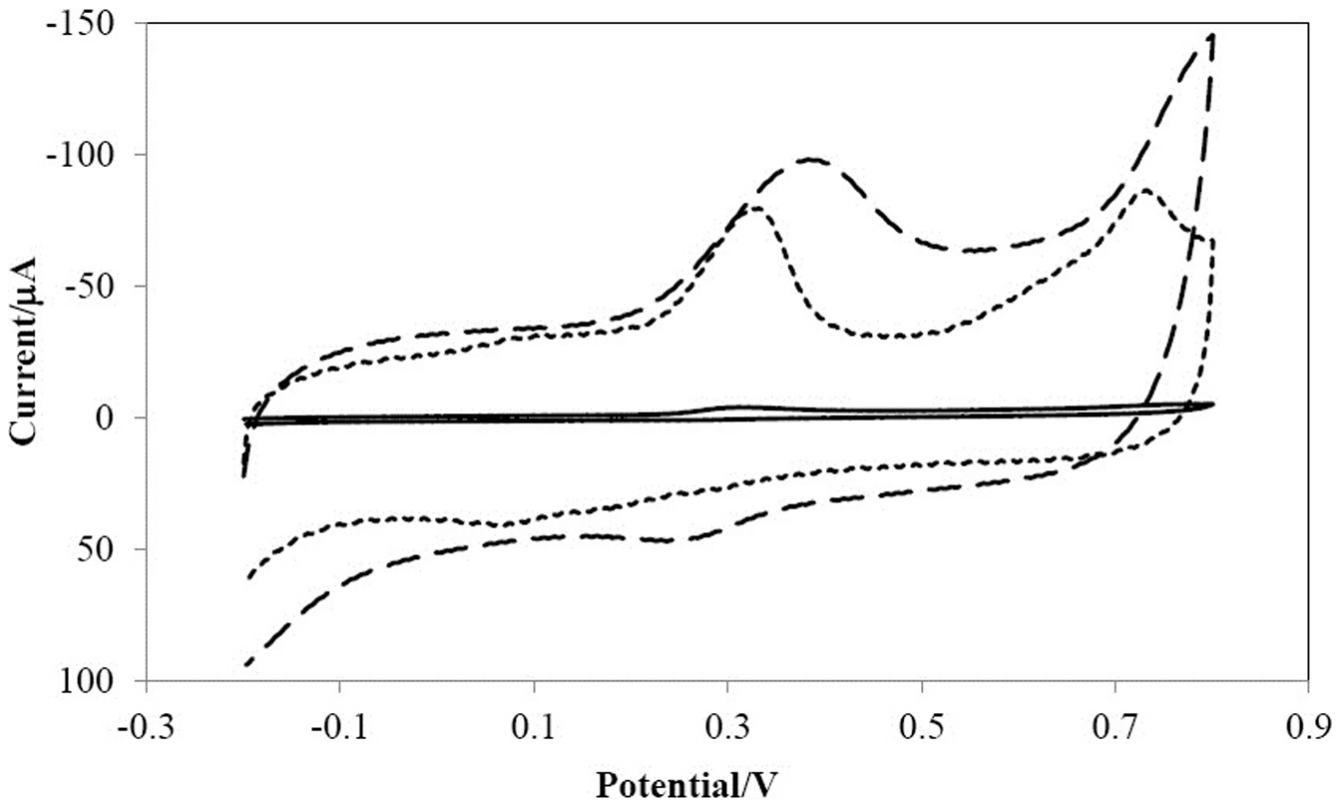
Cyclic voltammograms of 2.0×10^−5^ mol L^−1^ myricetin in 0.1 mol L^−1^ phosphate buffer (pH 3.5) on the different electrodes: the bare GCE (a), MWCNTs/GCE (b) and AuNPs/en/MWCNTs/GCE (d); scan rate 0.1 V s^−1^.

Fig. 3 shows cyclic voltammograms of myricetin on different kinds of modified electrodes. Myricetin shows a week oxidation peak at bare GCE (a), due to the weaker adsorption and slower electrochemical reaction rate on GCE surface. While, there are well-defined redox peaks on the MWCNTs/GCE (b) and AuNPs/en/MWNTs/GCE (c) in 0.1 mol L^−1^ phosphate buffer solution (pH 3.5). The peak currents are significantly higher and more reversible on the AuNPs/en/MWCNTs/GCE. The position of anodic (E_pa_) and cathodic peak potentials (E_pc_) were at approximately 0.379 V and 0.259 V (vs. Ag/AgCl) respectively and the ratio of i_pa_/i_pc_ was smaller than 1.0, which showed that electrode reaction was almost irreversible. AuNPs and MWCNTs can enhance the rate of electron-transfer and make more myricetin available in the electrochemical reaction due to their accumulation and catalytic ability.

### Calculation of microscopic surface area

The microscopic area of the AuNPs/en/MWCNTs/GCE was calculated by cyclic voltammetry using 1 mmol L^−1^ 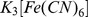 as a probe at different scan rates. For a reversible process, the Randles-Sevcik formula (eqn. 1) has been used [25]: 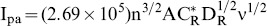
(1)where I_pa_ (A) refers to the anodic peak current, n; the number of electrons, A (cm^2^); surface area of the electrode, D_R_ (cm^2^ s^−1^) is diffusion coefficient, C_0_ (mol cm^−3^); concentration of 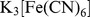 and ν (V s^−1^) is scan rate. For 1 mmol L^−1^ 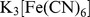 in the 0.1 mol L^−1^ KCl, n is equal to 1 and D_R_ = 7.6×10^−6^ cm^2^ s^−1^, then from the slope of the i_pa_ - ν^1/2^ equation, the microscopic area can be calculated. On the AuNPs/en/MWCNTs/GCE, the microscopic area was 0.0135 cm^2^.

### Effect of Scan Rate

The effect of scan rate on the electrochemical response of 2.0×10^−5^ mol L^−1^ myricetin was studied in the range of 20 to 200 mV s^−1^. There was a good linear relationship between the anodic peak current and scan rate (v). The regression equation was 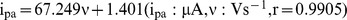, showing mass transfer controlled by adsorption process [25], [26]. The redox peak potentials shifted with increasing scan rate and also the peak-to-peak separation became larger, which demonstrated that electrochemical reaction gradually extended to more irreversibility. The peak potential was linear with logarithmic value of scan rate as 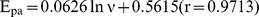 and 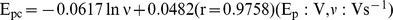. According to the equations (2, 3) [27], the electron-transfer coefficient α and n_a_ were estimated to be 0.50 and 1.0 respectively. 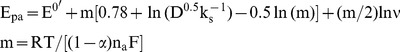
(2)

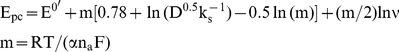
(3)


According to equation (4) [28], the slope of I_pa_-ν plot for myricetin in irreversible redox reactions is proportional to the total number of electrons transferred (n). By using chronocoulometry, the value of Q_f_ was calculated as 0.003 C. Finally, the total number of electrons in the oxidation step was determined as 1.95≈2.0. 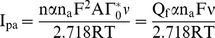
(4)


### Effect of pH

The effect of pH on the oxidation current of myricetin has been studied at 2×10^−5^ mol L^−1^ myricetin using phosphoric acid as the supporting electrolyte. By changing pH from 2.0 to 9.0, the anodic peak current shifted in the negative direction. There was a linear relationship between the anodic peak potential and pH value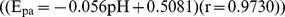. According to the slope of 56 mV pH^−1^, it can be deduced that the number of electrons and protons transferred were equal in the electrochemical reaction. With increasing pH value, the peak current increased up to pH 3.5, and then decreased at pH>3.5. This is due to the proton involved in electrochemical reaction. In pH>7.0 because of electrostatic repulsion between AuNPs and myricetin, the sensitivity decreased remarkably. Fig. 1C shows the recommended mechanism for electrochemical reaction of myricetin on the surface of AuNPs/en/MWCNTs/GCE modified electrode.

### Effect of accumulation potential

The effect of accumulation potential on the oxidation peak current of myricetin was examined in the range of 0.40 to −0.70 V. The oxidation peak current increased by changing accumulation potential from 0.40 V to 0.0 V and was nearly independent to E_acc_ from −0.20 to −0.70 V. Therefore, accumulation potential of 0.0 V was selected as the optimum value for determination of myricetin on the surface of modified electrode.

### Effect of accumulation time

**Figure 4:**
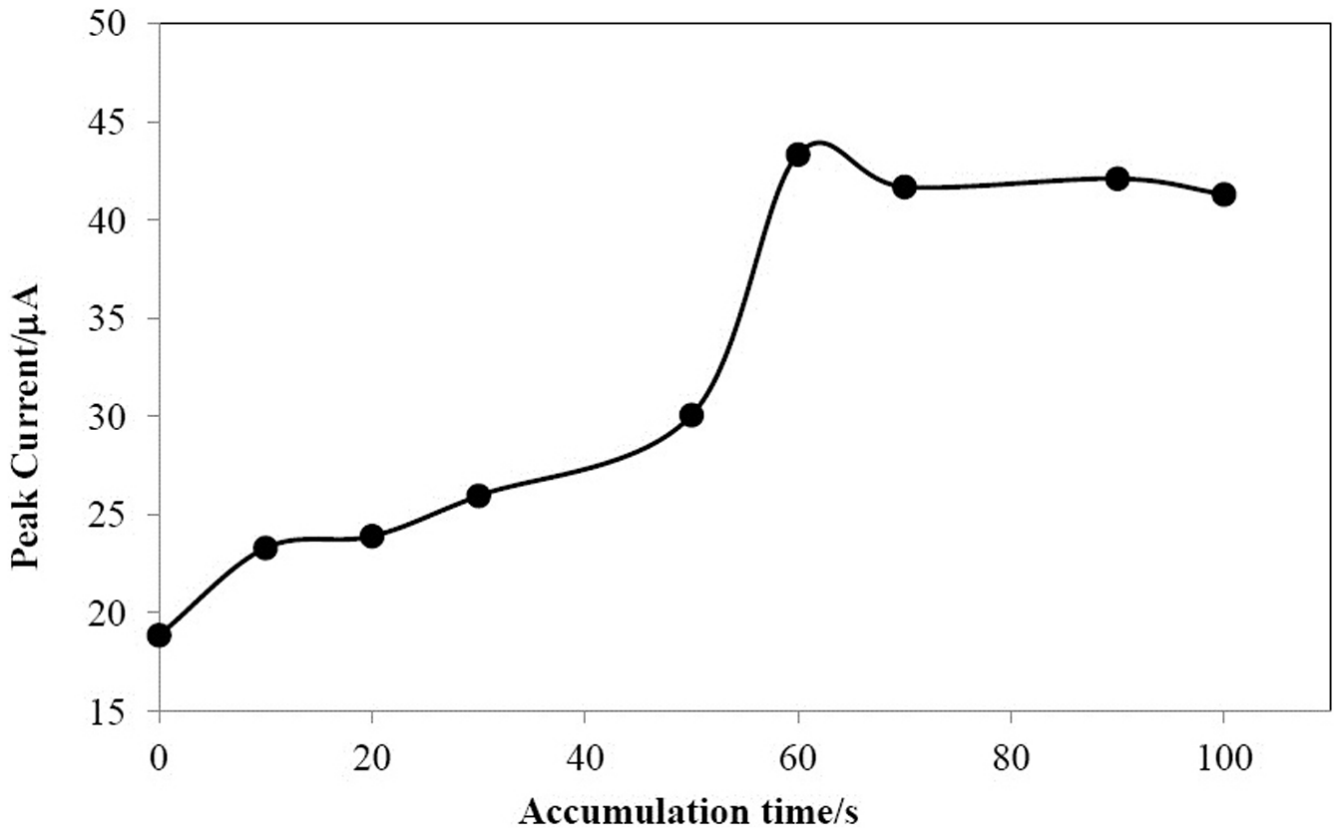
The dependence of accumulation time on the oxidation peak current of 2.0×10^−5^ mol L^−1^ myricetin in 0.1 mol L^−1^ phosphate buffer (pH 3.5) and E_acc_ = 0.0 V on the surface of AuNPs/en/MWCNTs/GCE.

The effect of accumulation time (t_acc_) on the oxidation peak current of myricetin was studied under accumulation potential of 0.0 V (Ag/AgCl) and pH 3.50. Increasing the accumulation time causes to an increase in the oxidation peak current and finally level off (t_acc_>60 s) due to the saturation of electrode (Fig. 4). Therefore, accumulation time of 60 s was selected for further studies.

### Calibration curve

In order to test the feasibility of the exploited method for the quantitative analysis of myricetin, the relationship between anodic peak current and concentration of myricetin was studied using cyclic voltammetry adsorptive stripping voltammetry (CVAdSV). Under the optimum parameters (pH 3.50, accumulation potential: 0.0 V, accumulation time: 60 s, scan rate: 0.1 V s^−1^), the calibration curve was linear in the range of 5.0×10^−8^ mol L^−1^ to 4.0×10^−5^ mol L^−1^ with three different equations. The regression equations were: 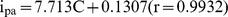 (0.05–2.0 µmol L^−1^), 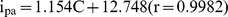 (2–15 µmol L^−1^) and 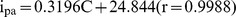 (15–40 µmol L^−1^) where, i_pa_ is anodic peak current (µA) and C is myricetin concentration (µmol L^−1^).

The sensitivity of a method is expressed by limit of detection (LOD) and limit of quantification (LOQ). The LOD and LOQ, established as the amounts for which the signal-to-noise-ratios were 3∶1 and 10∶1, respectively, were 1.20×10^−8^ mol L^−1^ and 4.02×10^−8^ mol L^−1^, respectively (Table 1). The relative standard deviations (n = 6) for 10 and 30 µmol L^−1^ myricetin were 2.2 and 3.6%, respectively, shows good reproducibility. The relative standard deviation for inter days operations (6 days) for 10 mM myricetin was 3.4%, shows good repeatability for the fabricated sensor. The AuNPs/en/MWCNTs/GC electrode can be stored in room temperature up to 3 weeks and can be used for about 50 cyclic voltammograms.

**Table 1:** The contrast with previous reports of LOD and LOQ for quantification of myricetin.

Component	Method	LOD/mol L^−1^	LOQ/mol L^−1^	Ref.
Myricetin	LC*	3.14×10^−7^	9.43×10^−7^	[14]
Myricetin	SPME-HPLC**	1.52×10^−7^	4.59×10^−7^	[15]
Myricetin	Electrochemical sensor	1.20×10^−8^	4.02×10^−8^	Present work

*Liquid Chromatography.

**Solid Phase Microextraction-High Performance Liquid Chromatography.

### Interference studies

The presence of other electroactive compounds is expected to affect considerably the analytical signal. The interfering effect of some representative compounds was studied in detail. Among these compounds - ascorbic acid, phenol, isoflavones, polysaccharides, aminoacids and metal salts were included because they are always present in nutrition foods, supplementary drugs, fruit juices and plant extracts. The effect of interferences at several molar ratios over myricetin on the measured analytical signal is given in Table 2. The tolerance limit was defined as the concentration, which give an error of ±10% in the determination of 1×10^−5^ mol L^−1^ myricetin. As it has shown, the fabricated electrochemical sensor is selective for determination of myricetin and suffers from some major interferences.

**Table 2:** Interference study for determination of 1.0×10^−5^ mol L^−1^ myricetin.

Species	Tolerance limits (C_species_/C_myricetin_)
Glucose, Fructose, Benzoic acid, Alanine, Asparagine, Glycine, Leucine, Proline, Serine, Theronine, K^+^, ClO_4_^−^, NO_3_^−^, PO_4_^3−^, HPO_4_^2−^, Cl^−^, Ca^2+^, Mg^2+^, Fe^3+^, Fe^2+^, SO_4_^2−^, CO_3_^2−^, Na^+^	>500
Ascorbic acid, Urea	200
Kaempferol	15
Morin	10

### Application

To investigate the possibility of using modified nanocomposite electrode for practical analysis, some fruit juices, black tea and green tea samples have been analysed by voltammetry after prior accumulation on the surface of nanocomposite electrode. The experimental results are listed in Table 3. As it has shown, recovery of the spiked concentrations are in the range of 101–104%, indicating that the modified electrode might provide a feasible alternative tool for determining of myricetin in natural products.

**Table 3:** Determination of myricetin in some kinds of tea and fruit juices (n = 3).

Sample	*C*_added_/mg g^−1^	*C*_found_/mg g^−1^	Recovery (%)
Black Tea	- - -	4.42±0.29	- - -
	0.79	5.26±0.34	104.50
Green Tea	- - -	27.18±1.25	- - -
	7.96	35.42±1.37	103.43
Gripe Juice	- - -	5.02±0.69	- - -
	3.80	8.85±1.65	101.06
Apple Juice	- - -	7.90±0.79	- - -
	3.80	11.80±2.21	102.53

## Conclusions

In this study, electrochemical behavior of myricetin was investigated on the AuNPs/en/MWCNTs/GCE in 0.01 mol L^−1^ phosphate buffer solution (pH 3.5), and optimization parameters including pH, accumulation potential, accumulation time and scan rate have been optimized. Under the optimized conditions, anodic peak current was proportional to the concentration of myricetin in the range of 5.0×10^−8^ to 4.0×10^−5^ mol L^−1^ with detection limit of 1.2×10^−8^ mol L^−1^. The fabricated electrochemical sensor displayed excellent characteristics, such as simplicity, economy, good sensitivity, selectivity, rapid analysis and wide detection range that offers good possibility for using as a sensor for analysis in real samples without pretreatment. The modified nanocomposite electrode was further applied to the determination of myricetin in tea and fruit juices with satisfactory results.

## References

[1] Chen S, Murray RW (1999) Electrochemical quantized capacitance charging of surface ensembles of gold nanoparticles. J Phys Chem B 103: 9996–10000.

[2] Li J, Yamada Y, Murakoshi K, Nakato Y (2001) Sustainable metal nano-contacts showing quantized conductance prepared at a gap of thin metal wires in solution. Chem Commun 21: 2170–2171. 10.1039/b106961f 12240096

[3] Haruta M (1997) Size- and support-dependency in the catalysis of gold. Catal Today 36: 153–166.

[4] Subramanian V, Wolf EE, Kamat PV (2003) Green emission to probe photoinduced charging events in ZnO−Au nanoparticles. Charge distribution and fermi-level equilibration. J Phys Chem *B* 107: 7479–7485.

[5] Welch CW, Compton RG (2006) The use of nanoparticles in electroanalysis: a review. Anal Bioanal Chem 384: 601–619. 16402180 10.1007/s00216-005-0230-3

[6] Iljima S (1991) Helical microtubules of graphitic carbon. Nature 354: 56.

[7] Wang YR, Hu P, Liang QL, Luo GA, Wang YM (2008) Application of carbon nanotube modified electrode in bioelectroanalysis. Chin J Anal Chem 36: 1011–1016.

[8] Downard AJ, Tan ESQ, Yu SSC (2006) Controlled assembly of gold nanoparticles on carbon surfaces. New J Chem 30: 1283–1288.

[9] Cruickshank AC, Downard AJ (2009) Electrochemical stability of citrate-capped gold nanoparticles electrostatically assembled on amine-modified glassy carbon. Electrochim Acta 54: 5566–5570.

[10] Scheidt HA, Pampel A, Nissler L, Gebhardt R, Huster D (2004) Investigation of the membrane localization and distribution of flavonoids by high-resolution magic angle spinning NMR spectroscopy. Biochim Biophys Acta 1663: 97–107. 15157612 10.1016/j.bbamem.2004.02.004

[11] Dajas F, Rivera F, Blasina F, Arredondo F, Echeverry C, et al (2003) Cell culture protection and *in vivo* neuroprotective capacity of flavonoids. Neurotox Res 5: 425–432. 14715446 10.1007/BF03033172

[12] Mira L, Fernandez MT, Santos M, Rocha R (2002) Interactions of flavonoids with iron and copper ions: A mechanism for their antioxidant activity. Free Radic Res 36: 1199–1208. 12592672 10.1080/1071576021000016463

[13] Liu IM, Liou SS, Cheng JT (2006) Mediation of beta-endorphin by myricetin to lower plasma glucose in streptozotocin-induced diabetic rats. J Ethnopharmacol 104: 199–206. 16203117 10.1016/j.jep.2005.09.001

[14] Zhu T, Bi W, Row K (2011) Extraction and determination of quercetin and myricetin from *chamaecyparis obtusa* by ionic liquids-based monolithic cartridge. Chin J Chem 29: 1759–1763.

[15] Kumar A, Malik AK, Tewary DK (2009) A new method for determination of myricetin and quercetin using solid phase microextraction–high performance liquid chromatography–ultra violet/visible system in grapes, vegetables and red wine samples. Anal Chim Acta 631: 177–181. 19084623 10.1016/j.aca.2008.10.038

[16] Sadik OA, Land WH, Wang J (2003) Targeting chemical and biological warfare agents at the molecular level. Electroanalysis 15: 1149–1159.

[17] Yang SL, Yang R, Li G, Qu LB, Li JJ, et al (2010) Nafion/multi-wall carbon nanotubes composite film coated glassy carbon electrode for sensitive determination of caffeine. J Electroanal Chem 639: 77–82.

[18] Yang SL, Qu L, Li G, Yang R, Liu C (2010) Gold nanoparticles/ethylenediamine/carbon nanotube modified glassy carbon electrode as the voltammetric sensor for selective determination of rutin in the presence of ascorbic acid. J Electroanal Chem 645: 115–122.

[19] Barbier B, Pinson J, Desarmot G, Sanchez M (1990) Electrochemical bonding of amines to carbon fiber surfaces toward improved carbon-epoxy composites. J Electrochem Soc 137: 1757–1764.

[20] Deinhammer RS, Ho M, Anderegg JW, Porter MD (1994) Electrochemical oxidation of amine-containing compounds: a route to the surface modification of glassy carbon electrodes. Langmuir 10: 1306–1313.

[21] Adenier A, Chehimi MM, Gallardo I, Pinson J, Vila N (2004) Electrochemical oxidation of aliphatic amines and their attachment to carbon and metal surfaces. Langmuir 20: 8243–8253. 15350099 10.1021/la049194c

[22] Melrnikova NB, Ioffe ID, Tsareva LA (2002) Reaction of bioflavonoids with copper (II) acetate in aqueous solution. Chem Nat Compd 38: 33–39.

[23] Safavi A, Ensafi AA (1991) Kinetic spectrophotometric determination of traces of sulphite. Anal Chim Acta 252: 121–126.

[24] Cruickshank AC, Tan ESQ, Brooksby PA, Downard AJ (2007) Are redox probes a useful indicator of film stability? An electrochemical, AFM and XPS study of electrografted amine films on carbon. Electrochem Commun 9: 1456–1462.

[25] Bard AJ, Faulkner LR (1980) Electrochemical methods, fundamentals and applications, Wiley & Sons, New York, pp. 218–219.

[26] Ensafi AA, Hajian R (2006) Determination of rutin in pharmaceutical compounds and tea using cathodic adsorptive stripping voltammetry. Electroanalysis 18: 579–585. 10.2116/analsci.24.1449 18997374

[27] Nicholson RS, Shain I (1964) Theory of stationary electrode polarography. Single scan and cyclic methods applied to reversible, irreversible, and kinetic systems. Anal Chem 36: 706–723.

[28] Bard AJ, Faulkner LR, Electrochemical methods, fundamentals and applications, Wiley & Sons, New York, 1980, pp. 525–526.

